# Partial root-zone drying combined with nitrogen treatments mitigates drought responses in rice

**DOI:** 10.3389/fpls.2024.1381491

**Published:** 2024-04-15

**Authors:** Minhua Zhao, Zhihong Gao, Chunyi Kuang, Xiaoyuan Chen

**Affiliations:** ^1^ Guangdong Provincial Key Laboratory of Utilization and Conservation of Food and Medicinal Resources in the Northern Region, College of Biology and Agriculture, Shaoguan College, Shaoguan, Guangdong, China; ^2^ Guangdong Engineering Technology Research Center for Efficient Utilization of Water and Soil Resources in North Region, College of Biology and Agriculture, Shaoguan College, Shaoguan, Guangdong, China; ^3^ School of Biology and Agriculture, College of Biology and Agriculture, Shaoguan College, Shaoguan University, Shaoguan, Guangdong, China

**Keywords:** RNA-seq, metabolomics, histological analysis, rice, osmotic stress, gene expression regulation, localized root-zone desiccation

## Abstract

Drought is a major stress affecting rice yields. Combining partial root-zone drying (PRD) and different nitrogen fertilizers reduces the damage caused by water stress in rice. However, the underlying molecular mechanisms remain unclear. In this study, we combined treatments with PRD and ammonia:nitrate nitrogen at 0:100 (PRD_0:100_) and 50:50 (PRD_50:50_) ratios or PEG and nitrate nitrogen at 0:100 (PEG_0:100_) ratios in rice. Physiological, transcriptomic, and metabolomic analyses were performed on rice leaves to identify key genes involved in water stress tolerance under different nitrogen forms and PRD pretreatments. Our results indicated that, in contrast to PRD_0:100_, PRD_50:50_ elevated the superoxide dismutase activity in leaves to accelerate the scavenging of ROS accumulated by osmotic stress, attenuated the degree of membrane lipid peroxidation, stabilized photosynthesis, and elevated the relative water content of leaves to alleviate the drought-induced osmotic stress. Moreover, the alleviation ability was better under PRD_50:50_ treatment than under PRD_0:100_. Integrated transcriptome and metabolome analyses of PRD_0:100_ vs PRD_50:50_ revealed that the differences in PRD involvement in water stress tolerance under different nitrogen pretreatments were mainly in photosynthesis, oxidative stress, nitrogen metabolism process, phytohormone signaling, and biosynthesis of other secondary metabolites. Some key genes may play an important role in these pathways, including *OsGRX4*, *OsNDPK2*, *OsGS1;1*, *OsNR1.2*, *OsSUS7*, and *YGL8*. Thus, the osmotic stress tolerance mediated by PRD and nitrogen cotreatment is influenced by different nitrogen forms. Our results provide new insights into osmotic stress tolerance mediated by PRD and nitrogen cotreatment, demonstrate the essential role of nitrogen morphology in PRD-induced molecular regulation, and identify genes that contribute to further improving stress tolerance in rice.

## 1 Introduction

Abiotic stresses (e.g., drought, salinity, heavy metals, or cold stress) can affect plant growth and development. Among them, drought is the most threatening and least controllable factor for crop growth (Seleiman et al., 2021). Drought is a natural phenomenon generated by a combination of climatic and environmental factors and generally occurs during periods of insufficient rainfall, ultimately leading to extensive crop damage and significant yield reduction (Al Azzawi et al., 2020). Currently, global warming, rainfall anomalies, and monsoon changes are driving the increasing incidence and stress of drought worldwide (Masih et al., 2014; Seleiman et al., 2021). The traditional approach to confront drought is to overcome the effects of water scarcity by using different nutrients, multiple solute levels, mulching, or deficit-regulating irrigation techniques. Most of these techniques are expensive, time-consuming, or require specific machinery (Iqbal et al., 2020). Regulated deficit irrigation is an optimization strategy that requires the crop to consciously tolerate a certain level of water deficit, sometimes resulting in decreased yield and significantly increased water use efficiency (WUE) (Yang et al., 2022; Zou and Kang, 2022). Partial root-zone drying (PRD) is a modified version of deficit irrigation and has been used to improve WUE by controlling drought (Jovanovic and Stikic, 2018). In this irrigation method, spatial separation of wet and dry roots is maintained throughout the growing season. The water stress generated in a part of the root zone produces the chemical signal abscisic acid (ABA), leading to partial stomatal closure, thus decreasing the transpiration rate and consequently increasing WUE (Ghafari et al., 2020; Iqbal et al., 2020).

Rice (*Oryza sativa* L.), a staple crop for more than half of the world’s population, grows in various environments, and rice yields directly impact food security worldwide (Ahmadi et al., 2014). However, rice is often subjected to abiotic stresses, such as drought, cold, and salinity, while growing. Because rice is a highly water-intensive crop, drought is a serious problem affecting rice production, and the lack of water can lead to significantly reduced productivity and yield (Quan et al., 2016; Hassan et al., 2023). Statistically, drought is particularly frequent in South Asia, Southeast Asia, and sub-Saharan Africa. India faced severe droughts in 2002 and 2009; the total rice production decreased by 210,000 tons in 2002 and 500,000 tons in 2009 (Simon and Shylaraj, 2017; Khan et al., 2021). Several studies have also demonstrated that rice root growth decreases under drought conditions, leaving a large scope for PRD technology to cope with drought in rice.

Population growth requires more land and water resources for food production. Improvements in arable land productivity and water use efficiency can be achieved by applying nitrogen fertilizer (Shahrokhnia and Sepaskhah, 2017). NH_4_^+^ promotes the growth of rice seedlings (Guo et al., 2007). Under water stress, increasing the NH_4_^+^ supply enhances stomatal conductance and CO_2_ conductance in rice leaves, thereby promoting the photosynthetic rate and maintaining photosynthesis (Li et al., 2012). Nitrogen uptake and transport mechanisms and soil microbial transformations make NO_3_^-^ the main form of available nitrogen for all crops, including rice (Sharma et al., 2022). The combination of NH_4_^+^ and NO_3_^-^ can improve the yield and quality of cabbage (Song et al., 2012), rapeseed (Li et al., 2021), and soybean (Raza et al., 2021) to varying degrees while mitigating the effects of water stress, whereas the optimal NH_4_^+^:NO_3_^-^ ratio varies depending on the crop species and environment. There is an obvious reciprocal effect between water and nitrogen. Water deficit inhibits nitrogen fertilizer uptake, while an excess causes soil nitrogen loss and reduces water and nitrogen use efficiency (Tang et al., 2010; Man et al., 2014). The water–nitrogen interaction can promote crop growth and improve yield and quality; water–nitrogen coupling technology is widely used in the cultivation of rice, wolfberry, and other crops (Ma et al., 2023). However, little research has been conducted on the synergistic effect between PRD and nitrogen. PRD improves crop nitrogen nutrition, optimizes nitrogen partitioning in plant organs, and increases the bioavailability of soil nitrogen (Ghafari et al., 2020; Liu et al., 2021). Previous studies in our laboratory have indicated that PRD can mitigate rice water stress with different nitrogen forms. Several studies have demonstrated that a single nitrogen form synergies with PRD to promote rice water use efficiency and alleviate water stress; however, the underlying molecular mechanisms remain elusive. This study compared the synergistic effects between PRD and two combinations of NH_4_^+^: NO_3_^-^ ratios of 0:100 and 50:50 via RNA sequencing and metabonomics analysis.

## 2 Materials and methods

### 2.1 Experimental design

**Figure 1:**
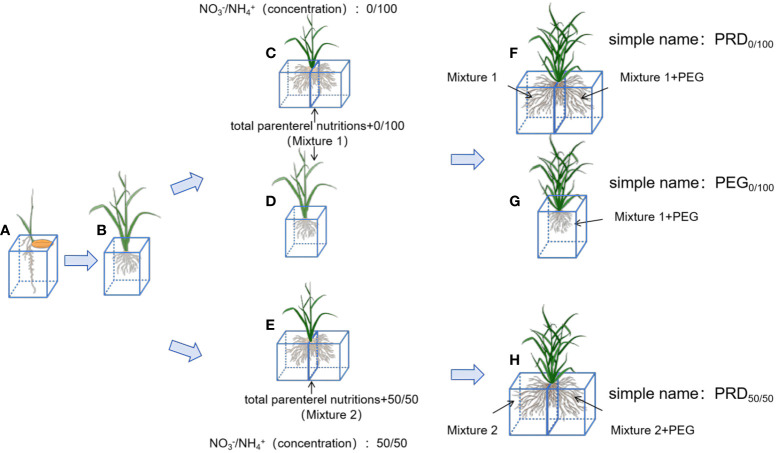
Schematic of the rice treatment. **(A)** Seeds of Meixiang sticky 2 were cultured using a full nutrient solution (**Supplementary Table S1**) until rice seedlings grew to four leaves and one heart. **(B)** Plants with uniform growth were selected and used to produce nutrient solutions with NH_4_^+^:NO_3_^-^-ratios of 0:100 (**Supplementary Table S2**) and 50:50 (**Supplementary Table S3**). Plants were cultured in whole-root **(D)**, PRD cultures **(C)** with NH_4_^+^:NO_3_^-^-ratios of 0:100 and PRD cultures **(E)** with NH_4_^+^:NO_3_^-^-ratios of 50:50 until obvious morphological differences appeared. Three treatments were applied to all plants: a NH_4_^+^:NO_3_^-^-ratios solution at 0:100 with PRD0:100 **(F)**, a NH_4_^+^:NO_3_^-^ solution at 0:100 with PEG0:100 **(G)**, and a NH_4_^+^:NO_3_^-^ solution at 50:50 with PRD50:50 **(H)**. Plants were incubated at 30°C during the day and 26°C at night, and the water stress lasted for 10 days.

The rice material used in this study was the excellent aromatic rice variety “Meixiangzhan No.2,” widely cultivated in southern China. After seed germination, the rice was cultured in a growth chamber at 32°C (12 h day/12 h night) until the four-leaf stage. Plants were divided into two experimental groups (all seedlings were used in triplicate) (**Figure 1**). In group 1, the NH_4_^+^:NO_3_^-^ ratio was 0:100. Roots were divided into two parts and inserted into two growth tubes: half of the roots were cultured with Hoagland’s nutrient solution, and the other half with a nutrient solution containing PEG-6000 [100 g/L (m/v)]. In group 2, the NH_4_^+^:NO_3_^-^ ratio was 50:50, and roots were split as described above. To minimize variation, plants were placed randomly, and each treatment comprised 12 rice plants. Ten days after treatment, mature leaves were collected, quickly frozen in liquid nitrogen, and stored at −80°C. Leaf tissues collected from these 12 plants were combined into three samples used for different further experiments: physiological parameter measurements, RNA-seq, and metabolomic analysis.

### 2.2 Physiological parameter measurements

The isolated leaves were soaked in distilled water for 4 h, and the saturation weight was recorded. The leaves were dried at 80°C until a constant weight was obtained, and the total dry weight was determined. The relative water content (RWC) was determined as follows: RWC = (fresh weight-dry weight)/(saturated weight-dry weight). The chlorophyll content was determined by UV spectrophotometry (Wei et al., 2014). Catalase (CAT), superoxide dismutase (SOD), and malondialdehyde (MDA) contents were determined using assay kits (Geruisi-bio, Suzhou, China).

### 2.3 Transcriptome analysis

Total RNA was extracted using the TRIzol reagent. The quality of the extracted RNA samples was examined using 1% agarose gel electrophoresis, NanoDrop 2000 (Thermo Fisher Scientific, Waltham, Massachusetts, USA), and Agilent 2100 Bioanalyzer (Agilent Technologies, Inc., Santa Clara, California, USA) (Li et al., 2019). Rice mRNA libraries were constructed using the standard protocols of the BGI Genome DNBSEQT7 platform. RNA-seq libraries were sequenced to obtain 150-bp paired-end reads. For sequence quality control, fastQ (0.21.0) was used to trim low-quality base pairs. The quality-filtered reads were aligned to the rice reference genome using Star (2.7.9a) (Kawahara et al., 2013). Only uniquely mapped reads were retained and analyzed for differential expression using DEseq2 (1.26.0) with a *p*-value <0.05 adjusted for fold change ≥ 2 and false discovery rate. Fragments per kilobase of transcript per million fragments mapped (FPKM) were calculated for each gene model using RSEM (Li and Dewey, 2011). Genes with at least 5 localized reads in 3 replicates and an average FPKM ≥ 1 were considered to be expressed.

### 2.4 Class-targeted metabolomic analysis

Class-targeted metabolomics analysis was used to identify and quantify the metabolites in rice samples. Metabolite-extracted tissues (100 mg) were individually ground thoroughly in liquid nitrogen and vortex-mixed by adding 500 mL of precooled 80% methanol. Samples were incubated under ice for 5 min and centrifuged at 15,000 × g and 4°C for 20 min. A portion of the supernatant was diluted to a final concentration containing 53% methanol. Then, the samples were transferred to new EP tubes and centrifuged at 15,000 × g and 4°C for 20 min. Finally, the supernatants were analyzed by LC-MS/MS using an ExionLC AD system (SCIEX, Framingham, MA, USA) and a QTRAP 6500+ mass spectrometer (SCIEX, Framingham, MA, USA) (Want et al., 2013).

### 2.5 Metabolite identification and quantification

Multireaction monitoring of experimental samples is based on an internal database (Zhang et al., 2022). The retention time has a narrow window and accurately identifies biochemicals using library entries (+/−0.005 amu), Q1 (parent ions), Q3 (daughter ions), and MS/MS forward and reverse scores between experimental data and authentic standards as criteria (Want et al., 2013). The data files generated by HPLC-MS/MS were processed using SCIEX OS (v1.4), and the peaks were integrated and corrected using the following parameter settings: minimum peak height, 500; signal-to-noise ratio, 5; and Gaussian smoothing width, 1. The area of each peak represents the relative content of the corresponding substance.

### 2.6 Integrated analysis of transcriptomic and metabolomic data

Gene ontology (GO) analysis (Wu et al., 2021) was performed on differentially expressed genes (DEGs) obtained from RNA-seq analysis to determine the biological functions of the genes, and *p* < 0.05 was significantly enriched to GO functions. Kyoto Encyclopedia of Genes and Genomes (KEGG) pathway enrichment analysis was performed to obtain the major pathways and routes involved in the differential genes, and *p* < 0.05 was significantly enriched to the pathways. Physiological parameters were statistically analyzed by R using Student’s *t*-test (*p* < 0.05). The identified metabolites were annotated using the KEGG database, the Human Metabolome Database, and the Lipid Map database. Principal component analysis and partial least squares discriminant analysis were performed using the metaX63 method (Wen et al., 2017). We used a one-way analysis of variance (*t*-test) to calculate statistical significance (*p*-value). Cluster analysis was performed on the differentially expressed metabolites (DEMs) to reveal representative trends within the two groups, and KEGG pathway enrichment analysis was used to reveal the pathways involved within the two DEM groups.

## 3 Results and discussion

### 3.1 Physiological changes in rice seedlings during PRD and osmotic stress treatment

**Figure 2:**
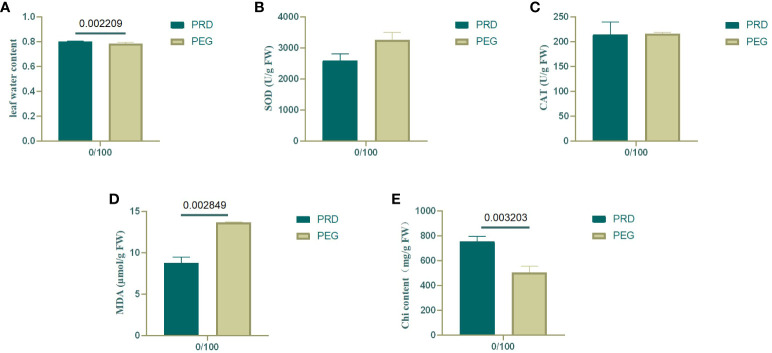
Changes in various physiological indexes of leaves under treatment with a nitrogen ratio of 0:100. **(A)** Water content of rice leaves; **(B)** SOD content of rice leaves; **(C)** CAT content of rice leaves; **(D)** MDA content of rice leaves; **(E)** Chlorophyll content of rice leaves. Dark green represents PRD_0:100_, and light green represents PEG_0:100_. The numbers above the columns represent the *p* values between the two treatments.

**Figure 3:**
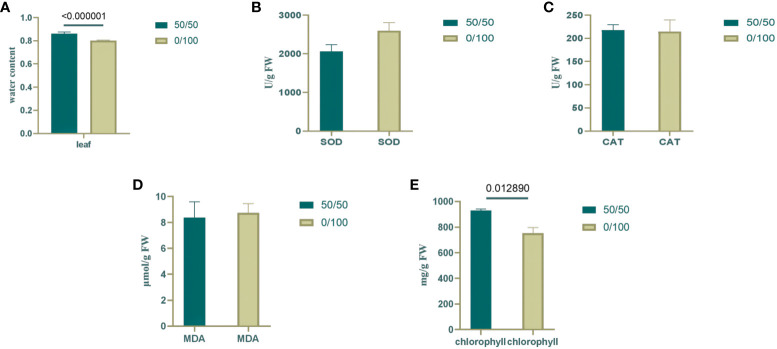
Changes in physiological indexes under different nitrogen ratios and PRD synergistic treatments. **(A)** Water content of rice leaves; **(B)** SOD content of rice leaves; **(C)** CAT content of rice leaves; **(D)** MDA content of rice leaves; **(E)** Chlorophyll content of rice leaves. Dark green represents PRD50:50, and light green represents PRD0:100. The numbers above the columns represent the p values between the two treatments.

At the onset of osmotic stress, the plant’s root system is restricted and struggles to absorb enough water to sustain growth. Under drought conditions, plants produce hydraulic, chemical, and phytohormonal signals transmitted to aboveground tissues to reduce water loss (Gao et al., 2020). The relative water content of leaves was measured to detect the effects of PEG and PRD treatments. Compared with the PEG treatment, the PRD treatment restored leaf water with a significant increase (**Figure 2A**), indicating that the PRD_0:100_ strategy alleviated osmotic stress or led to osmotic stress tolerance. Additionally, leaf water content was significantly higher under the PRD_50:50_ treatment compared with PRD_0:100_ (**Figure 3A**), indicating that different nitrogen treatments can affect the extent to which PRD restores water content.

Under osmotic stress, plants accumulate reactive oxygen species (ROS) and activate their antioxidant defense system in response to this oxidative damage. SOD catalyzes the conversion of superoxide anions from ROS into less harmful substances, whereas CAT breaks down hydrogen peroxide. By increasing the activity of SOD and CAT, plants can scavenge excess ROS, thereby protecting cells from damage (Tian et al., 2022). In the 0:100 nitrogen treatment, PEG-treated leaves had relatively high SOD activity, and the PRD treatment reduced SOD activity; however, catalase activity was stable (**Figures 2B, C**). Leaf SOD was relatively higher under PRD0:100 treatment compared with PRD50:50 (**Figures 3B, C**). Although the MDA content was also relatively high in PEG-treated leaves, indicating greater membrane lipid damage (**Figure 2D**), PRD treatment significantly reduced the MDA content. The MDA content of leaves under PRD50:50 treatment was relatively lower than with PRD0:100 (**Figure 3D**). These results indicate that PRD treatment could respond to osmotic stress by altering antioxidant enzymes and that different nitrogen treatments also affect the PRD regulation of antioxidant enzymes and attenuation of membrane lipid peroxidation.

Osmotic stress signaling also regulates photosynthesis, carbohydrate production, and energy metabolism. The chlorophyll content of PEG-treated leaves was significantly lower under the 0:100 treatment, and PRD treatment significantly increased the chlorophyll content, that PRD stabilized leaf photosynthesis (**Figure 2E**). Leaf chlorophyll content was lower and decreased significantly under PRD_0:100_ treatment compared to PRD_50:50_ (**Figure 3E**).

Overall, the synergistic treatment of PRD and nitrogen attenuated PEG-induced osmotic stress damage in plants. It could alleviate the osmotic stress damage caused by drought by enhancing SOD activity in leaves, accelerating the scavenging of ROS accumulated by osmotic stress, reducing the degree of membrane lipid peroxidation, stabilizing photosynthesis, and increasing the RWC of leaves. A previous study with the 50:50 nitrogen ratio treatment demonstrated that PRD can greatly alleviate the damage caused by osmotic stress. Similarly, PRD_50:50_ displayed a better trend than PRD_0:100_ in all physiological indices. Therefore, this mitigation effect is also related to the nitrogen form. In summary, the mitigation effect of PRD under a nitrogen ratio of 50:50 treatment is relatively better.

### 3.2 Analysis of DEGs and DEMs under synergistic treatment with nitrogen and PRDs

**Figure 4:**
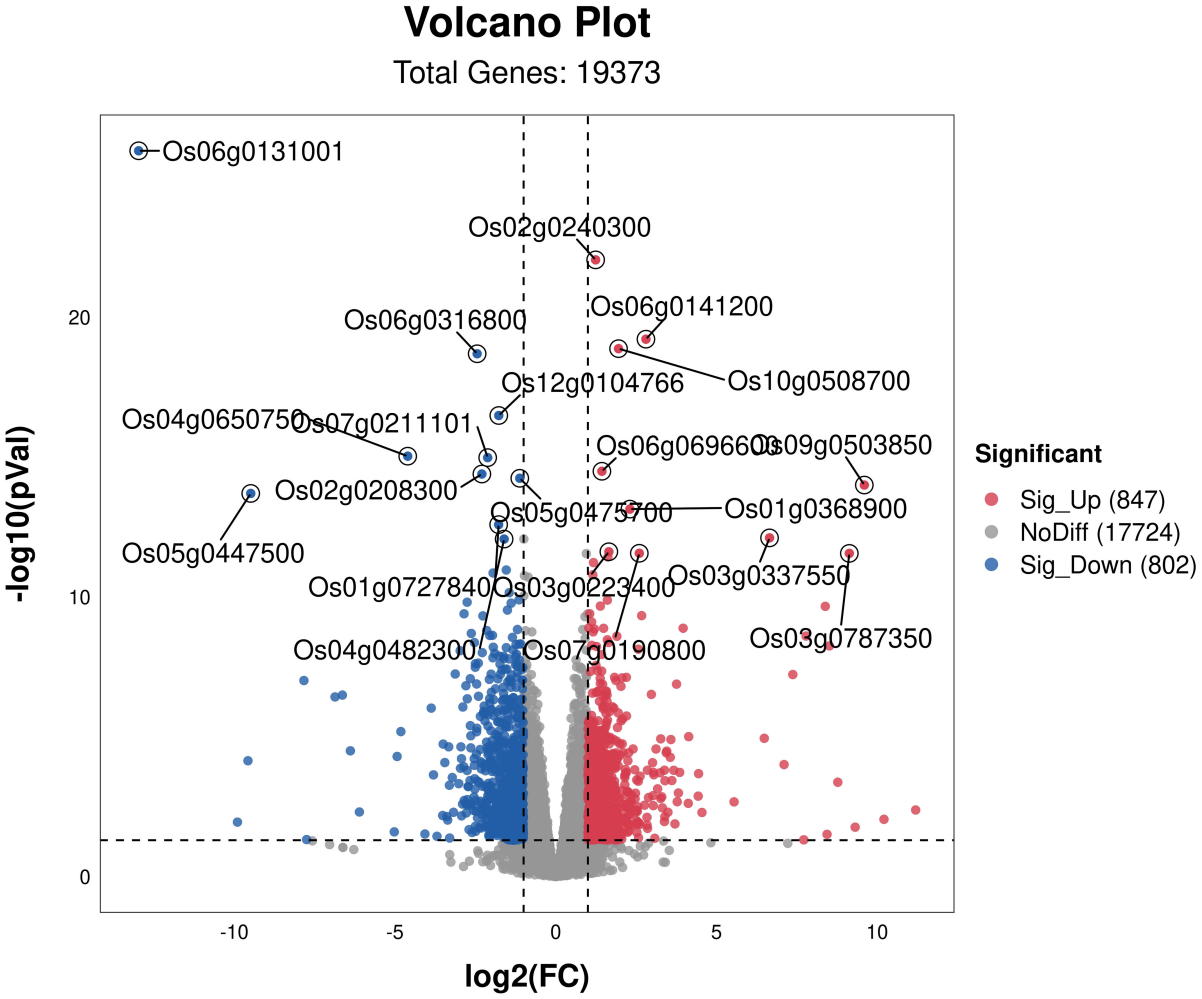
Volcanic map of DEGs for different treatments. DEGs in PRD_50:50_ vs PRD_0:100_ with *p*-value <0.05 and log2 (FC) ≥2. Red dots represent genes significantly upregulated in the comparison group, blue dots represent genes significantly downregulated in the comparison group, and gray dots represent genes that were not significantly regulated. The top 20 significantly regulated genes are marked in the figure.

To investigate the effects of osmotic stress mitigation in rice under different nitrogen ratios and PRD synergistic treatments, we performed RNA-seq sequencing and quasi-targeted metabolomics analysis. Six samples were sequenced by RNA-seq, yielding an average of 43325055.33 clean reads per sample, corresponding to 6487068375 clean bases (**Supplementary Table S4**). We identified 19,373 DEGS under PRD_50:50_ and 1,649 DEGs under PRD_0:100_, of which 847 were upregulated and 802 downregulated (**Figure 4**).

**Figure 5:**
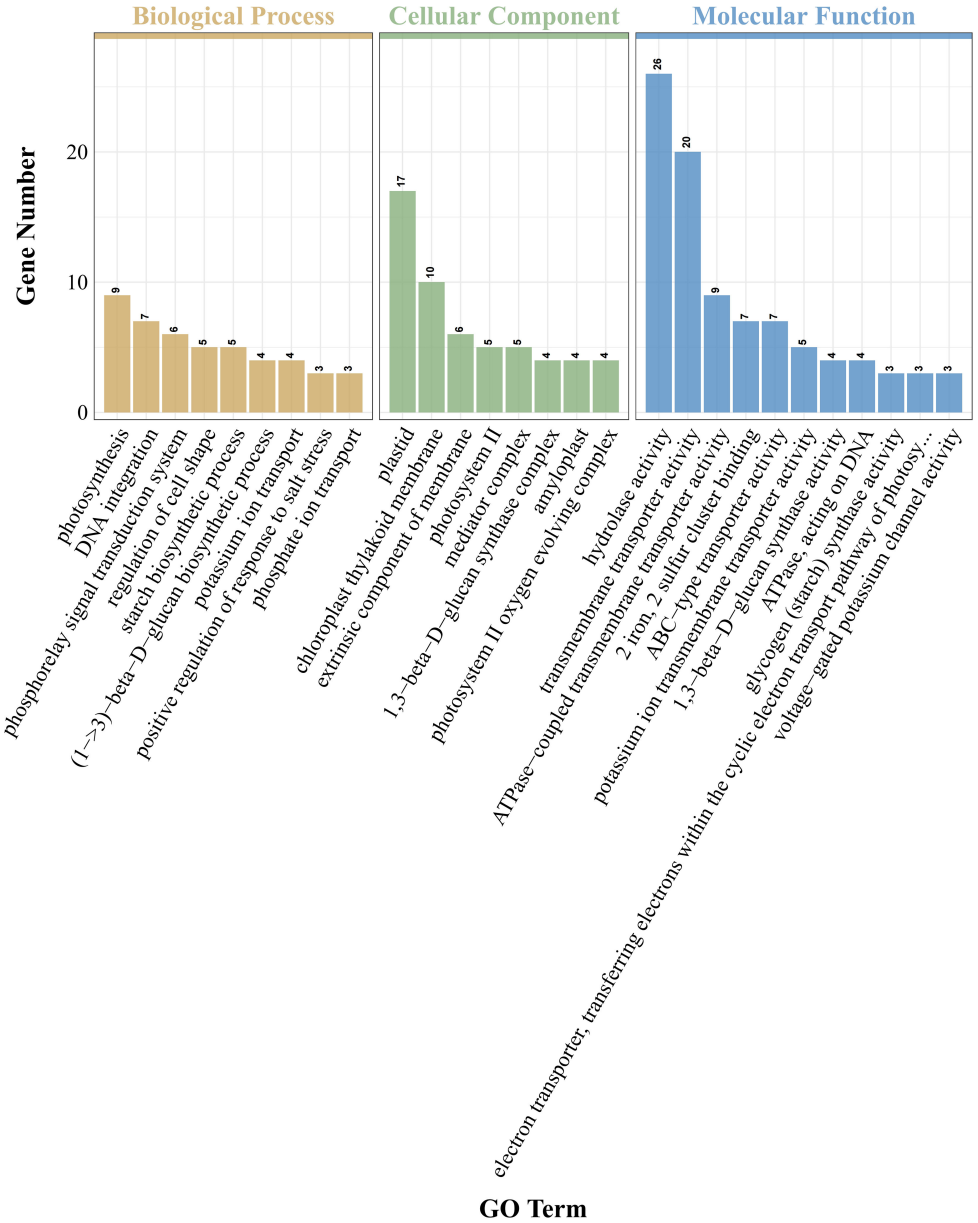
DEG GO analysis for PRD_50:50_ and PRD_0:100_ treatments. GO analysis was performed based on DEGs in PRD_50:50_ vs PRD_0:100_ and classified into three types of items, with the horizontal coordinate being the GO items annotated and the vertical coordinate being the number of genes.

GO analysis of DEGs indicated that GO items enriched in PRD_50:50_ and PRD_0:100_ treatments included biological processes, cellular composition, and molecular function (**Figure 5**). In the bioprocess category, DEGs in the PRD_50:50_ and PRD_0:100_ treatment groups were mainly distributed in the phosphotransfer signaling system, starch biosynthesis process, photosynthesis, glutamine metabolism process, glycogen biosynthesis process, and cytokinin response and signaling pathways. In contrast, in the cellular composition classification, DEGs in the PRD_50:50_ and PRD_0:100_ treatment groups were mainly concentrated in components with membranes, photosynthetic systems, chloroplasts, and transcription factor complexes. In the molecular function category, DEGs in the PRD_50:50_ and PRD_0:100_ treatment groups were predominantly distributed in transmembrane transport proteins and enzyme activities. Molecular function entries in the PRD_50:50_ and PRD_0:100_ treatments were the most numerous of the three entry species, and molecular functions related to defense responses, oxidative stress, and photosynthesis were the most affected by the PRD_50:50_ and PRD_0:100_ treatments.

**Figure 6:**
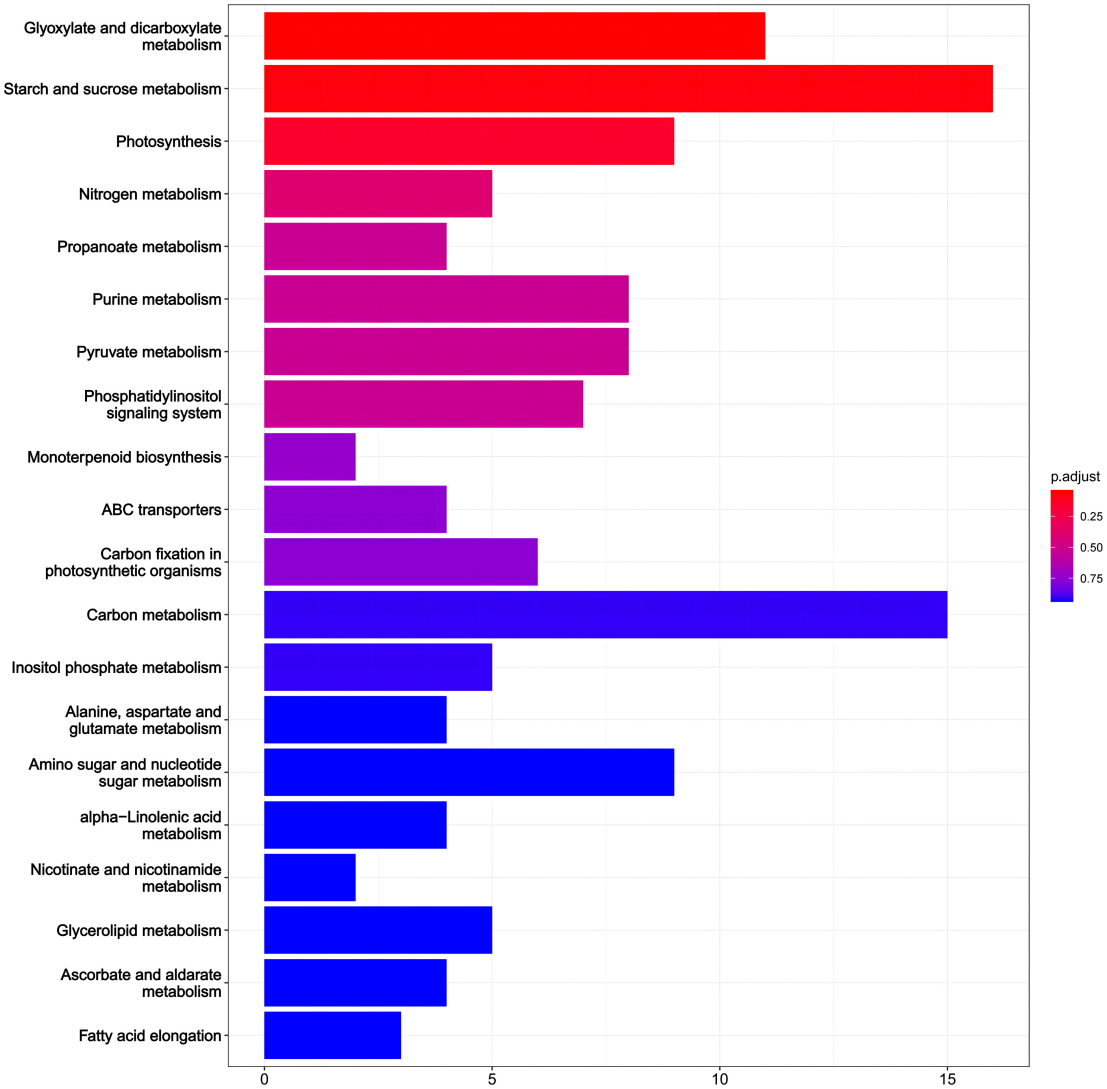
KEGG analysis of DEGs in PRD_50:50_ and PRD_0:100_ treatments. KEGG analysis based on DEGs in PRD_50:50_ vs PRD_0:100_, with the number of genes in each pathway in the horizontal coordinate and the pathway annotated in the vertical coordinate. The top 20 results are displayed according to the size of the *p*-values.

Additionally, we performed KEGG pathway analysis on DEGs (**Figure 6**). 210 DEGs in PRD50:50 vs PRD0:100 were assigned to 102 KEGG pathways. Categorizing the annotation results by pathway type, we observed that the pathways of DEGs were phased with metabolic and biosynthetic pathways and phytohormone signaling. The analysis indicated that the DEGs differed significantly in photosynthesis, nitrogen metabolic processes, phytohormone signaling, and biosynthesis of other secondary metabolites. From these metabolic pathways, we identified key genes that were differentially expressed in rice leaves after synergistic treatment with nitrogen forms and PRDs.

In KEGG enrichment analyses, pathways with p ≤ 0.05 were considered to significantly change in drought response under PRD50:50 and PRD0:100 treatments. **Figure 5** illustrates the top 20 enriched KEGG pathways. In the PRD50:50 vs PRD0:100 treatment group, pathways between DEGs included: glyoxylate and dicarboxylic acid metabolism, with 7 downregulated and 4 upregulated genes in PRD0:100; starch and sucrose metabolism, with 4 downregulated and 12 upregulated genes in PRD0:100; photosynthesis, with 7 downregulated genes in PRD0:100; nitrogen metabolism, with 2 genes downregulated and 3 upregulated genes in PRD0:100; propionic acid metabolism, with 3 downregulated and 1 upregulated gene in PRD0:100; purine metabolism, with 5 downregulated and 3 upregulated genes in PRD0:100; pyruvate metabolism, with 3 downregulated and 5 upregulated genes in PRD0:100; and the phosphatidylinositol signaling system, with 1 downregulated and 6 upregulated genes in PRD0:100 (**Supplementary Table S5**).

**Figure 7:**
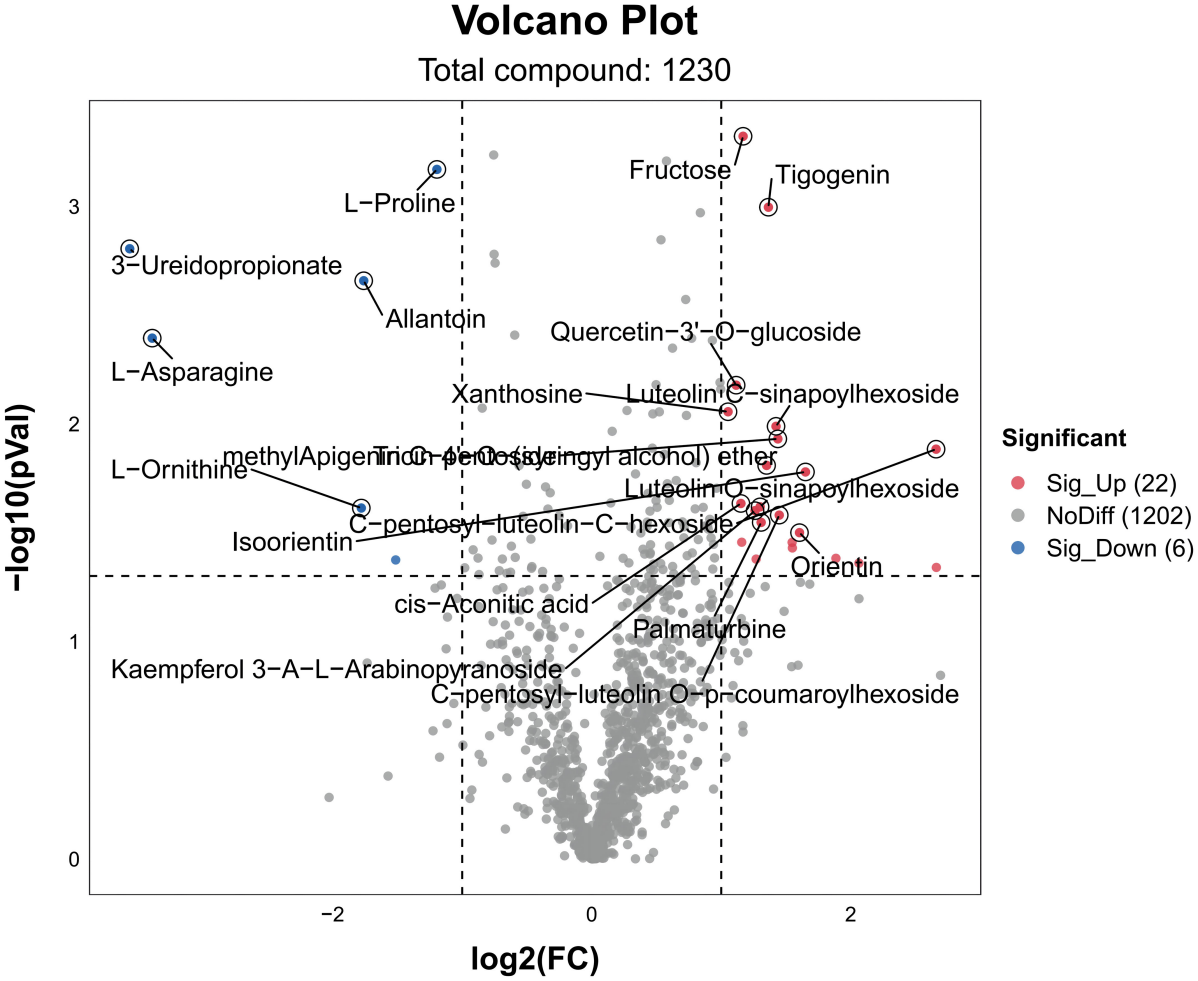
Volcanic maps of DEMs for different treatments. DEMs in PRD_50:50_ vs PRD_0:100_. Red dots are metabolites significantly upregulated in the comparison group, blue dots are metabolites significantly downregulated in the comparison group, and gray dots are metabolites that were not significantly affected in the comparison group. The top 20 DEMs are labeled in the figure.

We performed metabolite analyses on rice samples to assess the overall metabolic effects of different nitrogen ratios and PRD synergistic treatments on rice seedling leaves under drought conditions. DEMs were considered significant when VIP > 1, *p* < 0.05, and log2 (FC) ≥ 1 or ≤ −1. A total of 1230 differential metabolites were detected between PRD_50:50_ and PRD_0:100_ treatments, of which 22 were upregulated and 6 downregulated (**Figure 7**).

**Figure 8:**
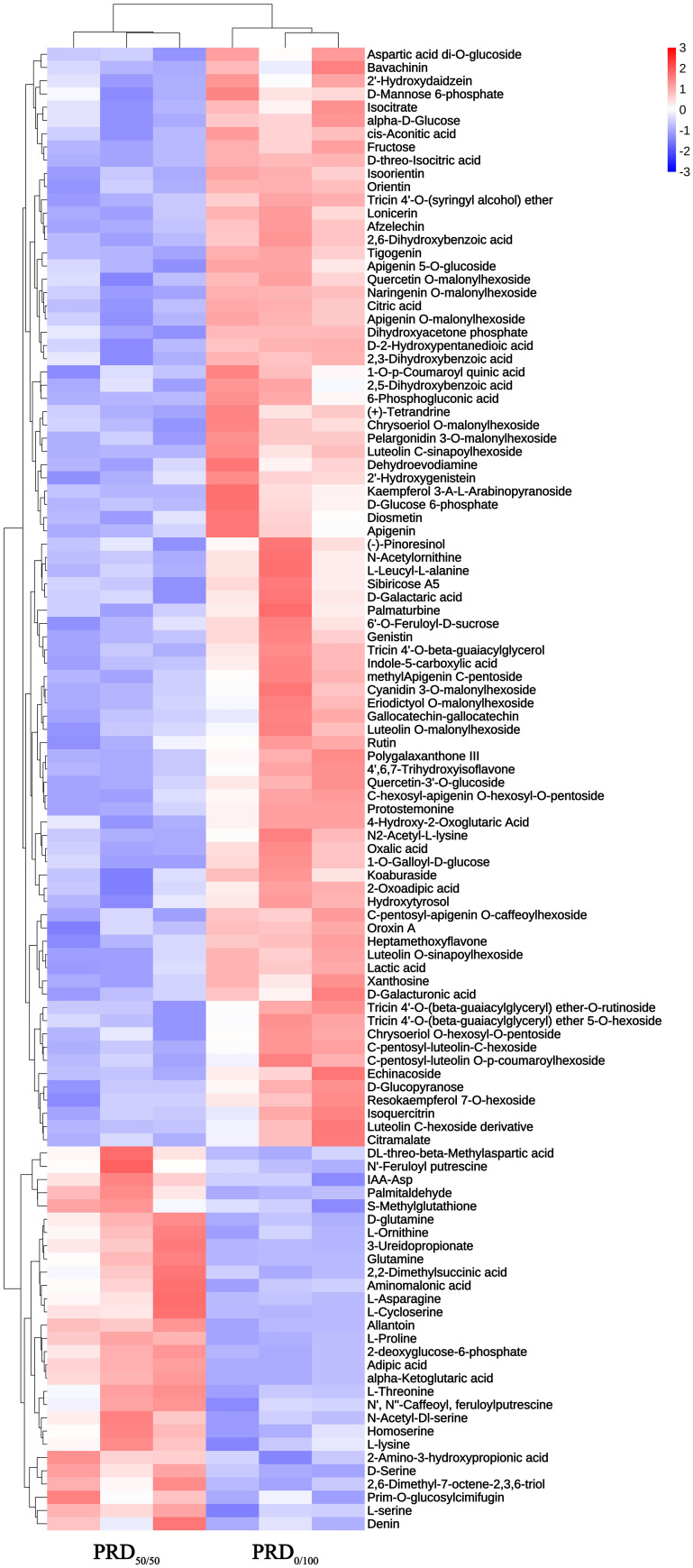
Hierarchical clustering analysis of DEMs in PRD_50:50_ and PRD_0:100_ treatment groups.

Hierarchical cluster analysis of the DEMs (**Figure 8**) yielded a picture of the differences in metabolic expression patterns between and within the two groups for the PRD_50:50_ and PRD_0:100_ treatment comparisons. The results indicated that DEMs had a significant aggregation tendency between the two treatment groups, and some amino acid substances, such as L -asparagine, D -glutamine, and L -threonine, were significantly upregulated under PRD_50:50_ and significantly downregulated under PRD_0:100_ treatments. A few amino acid substances, saccharides, and secondary metabolites were significantly upregulated in the PRD_0:100_ treatment group and significantly downregulated in the PRD_50:50_ treatment group.

**Figure 9:**
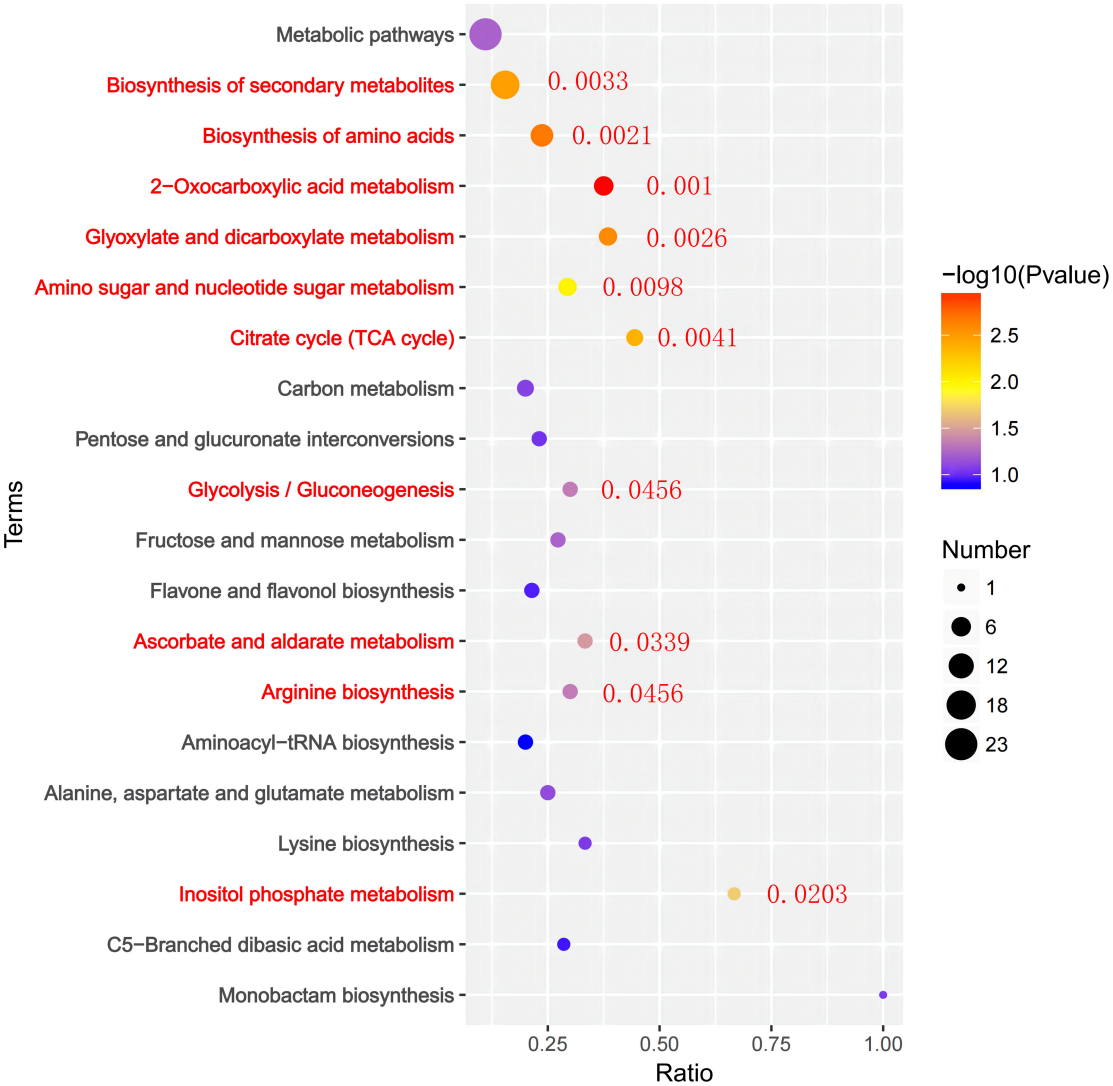
KEGG enrichment analysis of DEMs under PRD_50:50_ and PRD_0:100_ treatments. The horizontal coordinate is the ratio of genes in each metabolic pathway, the vertical coordinate is the metabolic pathway annotated to, and the size of the circle represents the number of genes. The top 20 KEGG terms are displayed according to the size of the *p*-value.

To identify the major pathways of rice seedling leaves in response to drought under nitrogen and PRD synergistic treatments, we mapped DEMs to KEGG pathways and obtained metabolic pathway enrichment results (**Figure 9**). DEMs were significantly assigned to 45 KEGG passages in PRD_50:50_ and PRD_0:100_ treatments, including 2 -oxocarboxylic acid metabolism, amino acid biosynthesis, glyoxylate and dicarboxylic acid metabolism, secondary metabolite biosynthesis, the citric acid cycle, aminoglycosan and ribonucleotide sugar metabolism, inositol phosphate metabolism-lipids, ascorbic acid and aldolase, glycolysis/glycolysis, and arginine biosynthesis.

### 3.3 Combining nitrogen forms and PRD protects rice leaves from drought by regulating the expression of oxidative stress-associated genes

**Figure 10:**
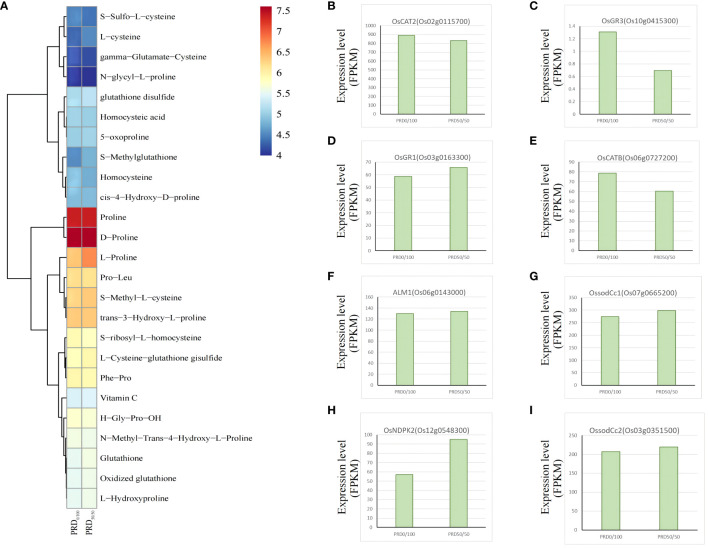
Key genes and metabolites involved in the antioxidant pathway. **(A)** Relative expression value of the major metabolites associated with the antioxidant pathway after performing -log10. **(B–I)** DEGs associated with the antioxidant pathway with FPKM expression values.

Various environmental stresses, including extreme temperature, salinity, drought, excessive light, and osmotic stress, induce elevated levels of ROS in various cellular compartments, mainly mitochondria, peroxisomes, and chloroplasts (Miller et al., 2007). ROS accumulate in large quantities in plant leaves, leading to severe stress responses and oxidative cell death. Excess ROS stimulate plant defense systems, such as ROS scavenging systems, antioxidant enzymes, glutathione, oxidoreductase, glutathione reductase (GRXs), and peroxidase to mitigate oxidative damage (Foyer and Noctor, 2011). Our study revealed several redox-related genes and metabolites that may have a central role in responding to redox processes (**Figure 10**), including the CC-type glutoxin family *OsGRX4* (Os01g0368900), *OsGRX29* (Os12g0538700), *OsGRX23* (Os11g0655900), and the nucleoside diphosphate kinase *OsNDPK2* (Os12g0548300), which may be involved in the plant’s defense mechanisms in response to ROS removal. All four genes were significantly upregulated in PRD_50:50_ vs PRD_0:100_ and were assigned to regulatory pathways in response to the H_2_O_2_ pathway and glutathione oxidoreductase activity (GO:0042542, GO:0097573) (**Supplementary Table S6**). Overexpressing *OsGRX4* in *Arabidopsis thaliana* resulted in increased levels of antioxidant enzymes (APX, POD, SOD, CAT), antioxidant molecules (ascorbic acid and GSH), and stress-responsive amino acids (cysteine and proline) in its transgenic lines, which exhibited longer roots, higher seed germination and survival, and consequently improved drought tolerance (Kumar et al., 2020). GRXs are glutathione-dependent and can regulate protein activity through reversible glutathionylation in the presence of glutathione reductase, detoxifying H_2_O_2_ by acting on antioxidant enzymes, such as peroxidase and ascorbic acid, thereby participating in redox signaling and ROS scavenging in response to oxidative stress (Kumar et al., 2020). We detected glutathione reductase *OsGR3* and *OsGR1* in PRD_50:50_ and PRD_0:100_ treatments, with a higher expression of *OsGR3* under the PRD_0:100_ treatment (**Figure 10C**) and a lower expression of *OsGR1* under the PRD_0:100_ treatment (**Figure 10D**). Catalase *OsCAT2* and *OsCATB* were more expressed under the PRD_0:100_ treatment (**Figures 10B, E**), and CAT activity in leaves was also higher in PRD_0:100_; however, none of those differences were significant (**Figure 3C**). The SOD *ALM1*, *OssodCc1*, and *OssodCc2* were all less expressed under the PRD_0:100_ treatment (**Figures 10F, G, I**); thus, SOD was more active under the PRD_50:50_ treatment (**Figure 2**). Some antioxidant molecules (ascorbic acid and GSH) and stress-responsive amino acids (cysteine and proline) displayed a higher expression in the PRD_50:50_ treatment group (**Figure 10A**). Photosynthesis is also a major source of ROS and H_2_O_2_, and chloroplasts, the main cellular component of photosynthesis, are highly susceptible to ROS damage. Many studies confirmed that *AtNDPK2* is involved in ROS signaling and oxidative stress management, and its overexpression induces the expression of various antioxidant genes, enhancing resistance to oxidative stress (Dorion and Rivoal, 2015). *OsNDPK2*, encoded by the WSL12 protein located in chloroplasts, was significantly upregulated under the PRD_50:50_ treatment (**Figure 10H**), and the oxidative status of *wsl12* mutant leaves was more severe than that of the wild type, which was involved in ROS scavenging by regulating the transcriptional levels of *APX1*, *APX2*, *AOX1a*, and *SODA1* (Ye et al., 2016).

### 3.4 DEGs involved in starch and sucrose metabolism

**Figure 11:**
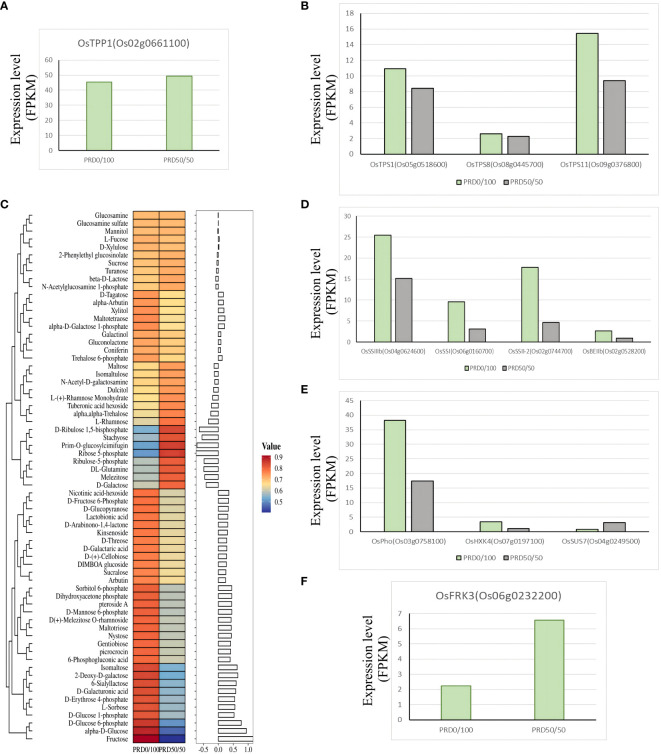
Key genes and metabolites involved in the starch and sucrose pathways. **(C)** Relative expression values of the major metabolites associated with the starch and sucrose pathways with *z*-score processing and the corresponding histogram for each metabolite is the log2FC value. **(A–F)** DEGs associated with the starch and sucrose pathways with the FPKM expression values.

Sucrose and starch are widely distributed in various plant tissues and are essential transport and storage substances for plant carbohydrates. Starch plays a key role in balancing growth and carbon assimilation, and its concentration increases during drought when it is converted into saccharides that act as osmoprotectants to alleviate leaf growth (Krasensky and Jonak, 2012; Thalmann and Santelia, 2017). The alginate pathway is essential in regulating the use and distribution of sucrose, harmonizing source–reservoir relationships, and enhancing the efficient use of carbohydrates (Han et al., 2023). Excess alginate has considerable potential to enhance abiotic stress tolerance in transgenic rice plants, which display higher levels of tolerance to salt, drought, and cold stresses compared to untransformed controls (Garg et al., 2002). We identified several alginate-6-phosphate phosphatases, *OsTPP1*, and alginate-6-phosphate highly expressed under the PRD_50:50_ treatment (**Figures 11A, C**), whereas the alginate-6-phosphate synthases *OsTPS1*, *OsTPS8*, *OsTPS11*, and alginate were all highly expressed under the PRD_0:100_ treatment (**Figure 11B**). Our results indicated that the starch and sucrose synthesis pathways were significantly enriched in the PRD_50:50_ vs PRD_0:100_ treatment group, and the expression levels of several genes were almost all significantly downregulated (**Supplementary Table S7**), including four genes related to 1,3 -β- D -glucan synthetase and genes related to starch synthase (*OsSSIIIb*, *OsSSI*, *OsSSII-2*, and *OsBEIIb*). Only fructose kinase *OsFRK3* (Os06g0232200), sucrose synthase *OsSUS7* (Os04g0249500), UTP-1-phosphoglucose uridylyltransferase (Os01g0264100), and α-amylase precursor (Os04g0403300) were significantly upregulated under the PRD_50:50_ treatment (**Figures 11D–F**). *OsFRK3* regulates starch accumulation and seed filling by affecting sugar metabolism (Zhang et al., 2023). Sucrose synthase (SUS) is a key enzyme necessary for the entry of sucrose, a product of leaf photosynthesis, into various metabolic pathways, and sucrose synthase *OsSUS7* (Os04g0249500) expression was significantly elevated under the PRD_50:50_ treatment (**Figure 11E**). OsSUS7 is central in the synthesis of starch in plants, improves plant resistance, and influences plant growth (Cho et al., 2011).

### 3.5 Nitrogen forms and PRD synergistic treatments protect rice leaves from drought by regulating the expression of phytohormone-related genes

**Figure 12:**
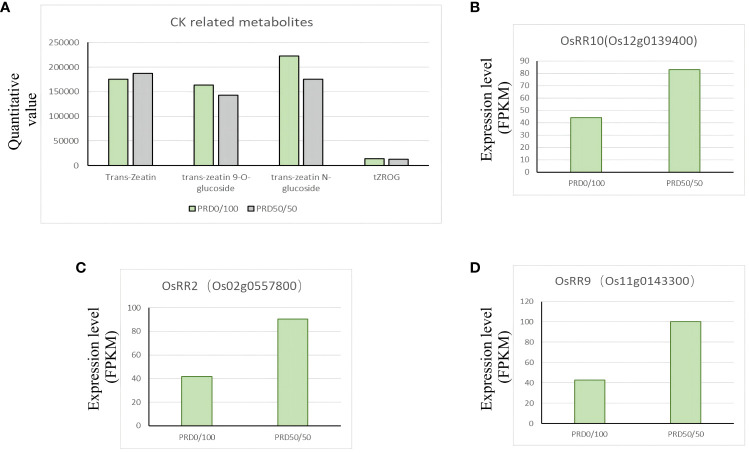
Changes in major plant hormones and key genes under different nitrogen ratios and PRD synergistic treatments. **(A)** Relative expression of CK-related phytohormones and metabolites. **(B–D)** DEGs related to phytohormone synthesis with FPKM expression values.

Plants can respond to damage caused by external stresses by regulating the hormone content in the plant body. Under drought, ABA can regulate the expression of drought-responsive genes, stomatal closure, ion homeostasis, and metabolic changes; it is the central phytohormone involved in drought responses (Li et al., 2016; Agurla et al., 2018; Waadt et al., 2022). Additionally, jasmonic acid, gibberellin, ethylene, and salicylic acid can regulate plant response to drought. We detected several phytohormones and metabolites related to phytohormones under PRD_0:100_ vs PRD_50:50_ treatment (**Supplementary Table S8**). Phytohormones, including growth hormone and its derivatives, jasmonic acid and its derivatives, ABA, gibberellin, salicylic acid, and cytokinins (CKs) and their derivatives, underwent a complex change, suggesting that the synergistic treatments of PRD and nitrogen ratios are important in response to osmotic stress, and different nitrogen ratios affect phytohormone biosynthesis and signal transduction differently. Growing evidence suggests that CK regulates drought acclimatization/adaptation in plants through a multistep phospho-reduction pathway (Zwack and Rashotte, 2015). *OsRR* genes are the primary genes that respond to CK, and *OsRR1* and *OsRR2*, among others, are induced by CK (Cho et al., 2022). Our GO analysis of DEGs under PRD_0:100_ vs PRD_50:50_ treatment identified four gene functions that were significantly enriched for the response to CK (GO:0009735) and CK-associated signaling pathways (GO:0009736) (**Supplementary Table S9**). Three type-A response regulators, *OsRR10* (Os12g0139400), *OsRR2* (Os02g0557800), and *OsRR9* (Os11g0143300), were significantly upregulated under the PRD_50:50_ treatment (**Figures 12B–D**), and several cytokinin-related derivatives, trans-zearalenin, trans-zearalenin N-glucoside, trans-zearalenin O-glucoside, and tZROG, were upregulated under the PRD_0:100_ treatment compared with the PRD_50:50_ treatment (**Figure 12A**). This observation suggests that cytokinin and cytokinin-related pathways function in response to drought under different nitrogen ratios.

### 3.6 Nitrogen forms and PRD synergistic treatments protect rice leaves from drought by modulating nitrogen uptake and use

**Figure 13:**
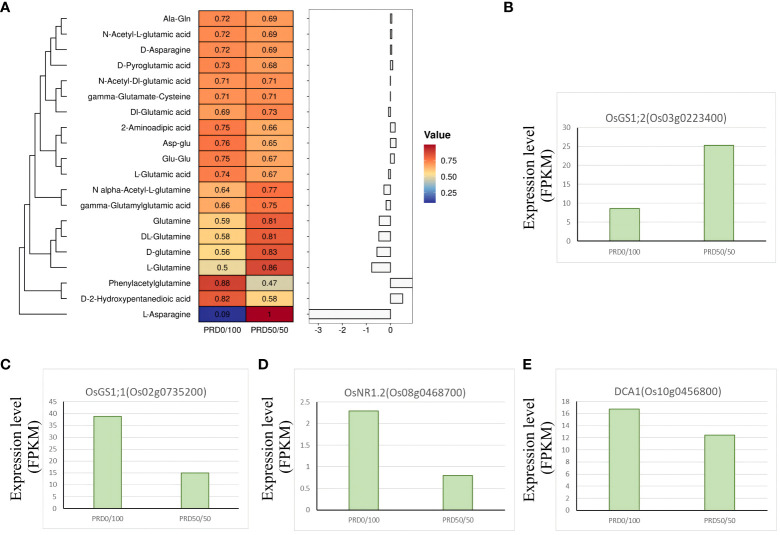
Key genes and metabolites involved in the nitrogen metabolism assimilation pathway. **(A)** Relative expression value of the major metabolites associated with the nitrogen metabolism assimilation pathway and the *z*-score processed. Each metabolite corresponds to a log2FC value in the histogram. **(B–E)** DEGs associated with the nitrogen metabolism assimilation pathway with FPKM expression values.

Nitrogen is one of the major nutrients governing crop yield, and glutamine synthetase (GS) is a key enzyme in the nitrogen metabolic pathway. Overexpressing GS increases the efficiency of nitrogen use in plants, thus contributing to plant growth and development and the response to abiotic stresses (Ohashi et al., 2018). Changes in the expression of GS genes before and after treatment with different nitrogen concentrations are closely related to the nitrogen content in plants (El Omari et al., 2010). Our KEGG analysis of DEGs was significantly enriched in two genes, OsGS1;2 (Os03g0223400) and OsGS1;1 (Os02g0735200), which are related to GS (**Figures 13B, C**). Nitrogen content was decreased in all aboveground parts of Osgs1;2 mutants, OsGS1;2 was significantly upregulated under the PRD50:50 treatment (**Figure 13B**), and levels of sugar metabolites, such as sucrose and glucose-6-phosphate, were disorganized (Ohashi et al., 2018). OsGS1;1 plays a key role in coordinating the entire metabolic network when rice uses ammonium salt as the main nitrogen source. Individual sugars, organic acids, and free amino acids, and the expression patterns in OsGS1;1-overexpressing plants, displayed significant changes when sufficient nitrogen fertilizer was provided in the environment, implying that OsGS1;1 plays a different role in rice nitrogen metabolism (Bao et al., 2014). While variable splicing of OsGS1;1 was induced under low nitrogen, OsGS1;1 expression was significantly higher under the PRD0:100 treatment (**Figure 13C**), affecting sugar and carbon metabolism, influencing nitrogen uptake and assimilation, and improving nitrogen use (Liu et al., 2022). This result suggests that OsGS1;1 is significantly induced in the absence of ammonium nitrogen and that the unbalanced carbon and nitrogen metabolic state and nitrogen translocation capacity may explain the extent of the drought response.

Plant nutrient use efficiency (NUE) decreases under osmotic stress, which may be due to reduced NO_3_^-^ transport, reduced nitrate reductase (NR) activity, or inhibition of other enzymes involved in nitrogen metabolism (Goel and Singh, 2015; Han et al., 2015; Meng et al., 2016). *OsNR2*, encoding an NADH/NADPH-dependent NR, controls the natural variation of NUE in rice and contributes to improving NUE in indica varieties and obtaining superior grain yield (Gao et al., 2019). Drought salt tolerance protein (DST) is a C2H2 zinc finger transcription factor in rice that negatively regulates osmotic stress tolerance and grain number by controlling genes involved in H_2_O_2_ and cytokinin homeostasis (Cui et al., 2015). Evidence suggests that DST controls NUE in rice and mediates the reprogramming of nitrogen assimilation under drought conditions through the direct regulation of *OsNR1.2* (Yi et al., 2022). When nitrate was sufficient, the loss-of-function mutant of *OsNR1.2* was more tolerant to osmotic stress, whereas *OsNR1.2* (Os08g0468700) was significantly less expressed under the PRD_50:50_ treatment (**Figure 13D**). *DCA1* (Os10g0456800), a DST-interacting protein that functions as a DST transcriptional coactivator, was also less expressed under the PRD_50:50_ treatment (**Figure 13E**). Products related to the nitrogen assimilation pathway, including glutamate, glutamine, asparagine, amino acids, and their derivatives that can be taken up by the plant, displayed relatively low levels under the PRD_50:50_ treatment (**Figure 13A**). Thus, the degree of nitrogen assimilation inhibition was higher under PRD_50:50_ than PRD_0:100_ (Han et al., 2022). This evidence suggests that the DST-OsNR1.2 module mediates the inhibition of nitrogen assimilation under osmotic stress and that the cotreatment with NH_4_^+^ and NO_3_^-^ under PRD treatment enhances rice survival in drought conditions by inhibiting nitrogen assimilation.

### 3.7 Nitrogen forms and PRD cotreatment protect rice leaves from drought by regulating photosynthesis

**Figure 14:**
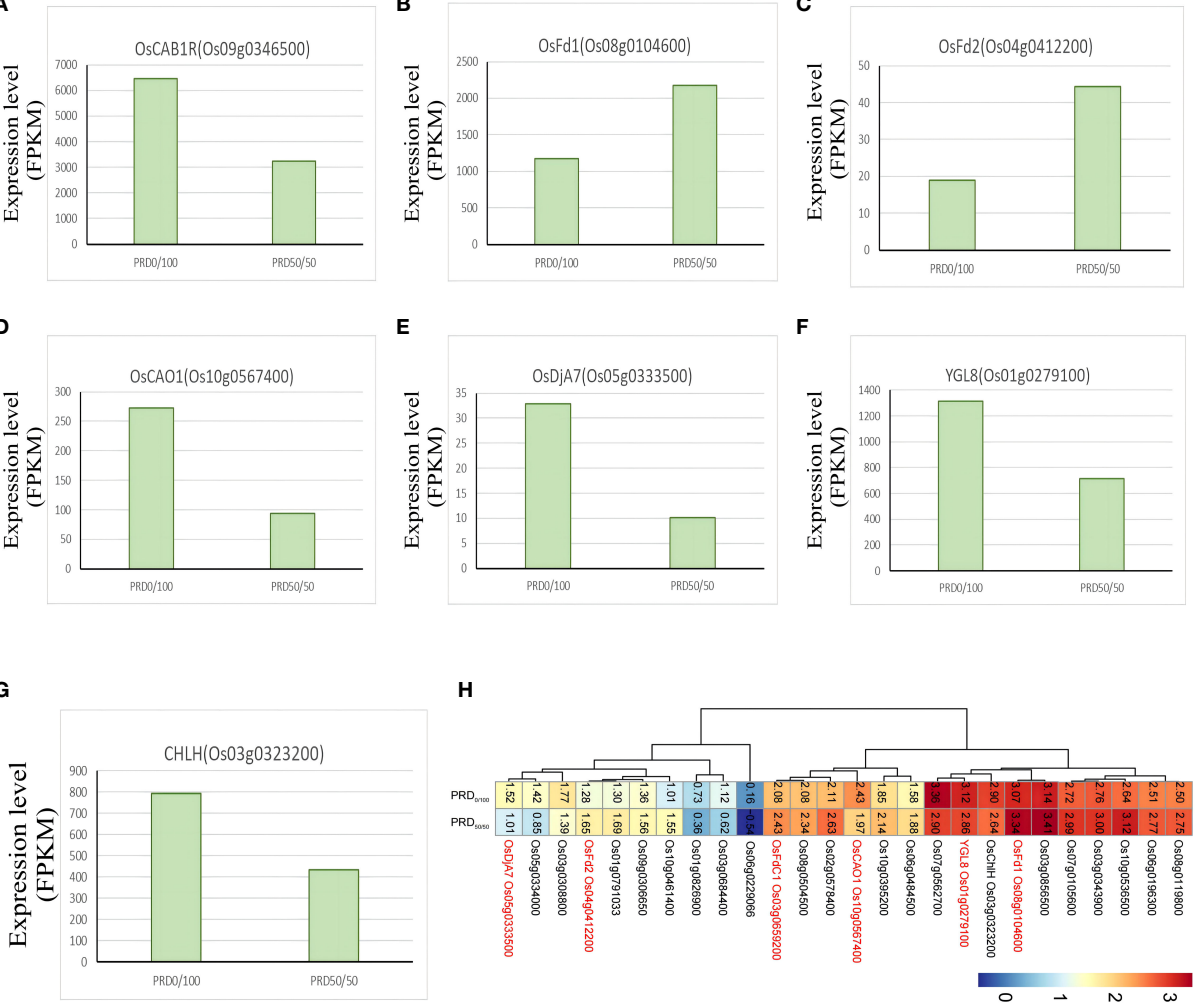
Key genes of the photosynthesis pathway. **(H)** FPKM values of all relevant genes in the photosynthesis pathway after -log10 cluster analysis. **(A–G)** DEGs related to the main photosynthesis with FPKM expression values.

Photosynthesis is the most basic life activity of plants and is very sensitive to drought; plants experience a decrease in the photosynthetic rate under drought adversity. Drought leads to the accumulation of ROS, which will injure the cell membrane, leading to chlorophyll, protein, and lipid degradation. We identified several genes involved in photosynthesis, with 27 genes annotated to functions related to photosynthetic pathways, chloroplasts, and photosystems (**Figure 14H**) (**Supplementary Table S10**). Ferredoxins (Fds) transfer electrons during photosynthesis and are important for the nitrogen assimilation pathway and chlorophyll metabolism (Tanaka and Tanaka, 2006). The ferredoxin *OsFd1* (Os08g0104600) was significantly upregulated under the PRD_50:50_ treatment (**Figure 14B**); it participates to the photosynthetic electron transport (He et al., 2020). The ferredoxin *OsFd2* (Os04g0412200) was also significantly upregulated in the PRD_50:50_ group (**Figure 14C**). *OsCAO1* (Os10g0567400) plays a major role in the synthesis of chlorophyll b; it is involved in light-mediated chlorophyll synthesis and was significantly upregulated in the PRD_0:100_ group (**Figure 14D**), enhancing photosynthesis rate by boosting chlorophyll content and alleviating Chl degradation and ROS accumulation under osmotic stress (Lee et al., 2005; Yang et al., 2015). *OsDjA7* (Os05g0333500) encodes a chloroplast localization protein, which is important for rice chloroplast development and differentiation and was significantly downregulated in the PRD_50:50_ group (**Figure 14E**). The expression of *OsCAB1R*, a gene closely related to chloroplast development, was also significantly downregulated in the PRD_50:50_ group (**Figure 14A**) (Zhu et al., 2015). *CHLH* (Os03g0323200) encodes the largest subunit of rice Mg-chelatase, which is essential in chlorophyll development and was significantly upregulated in the PRD_0:100_ group (**Figure 14G**) (Goh et al., 2010). Magnesium protoporphyrin IX monoester cyclase (MgPME) cyclase is an essential enzyme involved in chlorophyll synthesis, and *YGL8* (Os01g0279100) was significantly upregulated in the PRD_0:100_ group (**Figure 14F**). The mutant of *YGL8* exhibited a distinct yellow-green leaf phenotype at the seedling, tillering, and spike stages, especially in young leaves and spikes, where the chlorophyll content in the leaves was reduced, and the chlorophyll a/b ratio was elevated. The chlorophyll content in the leaves gradually returned to normal at the filling stage, and the leaves turned green (Kong et al., 2016). Many genes are involved in plant chlorophyll synthesis, catabolism, and signaling regulation, and mutations in any of these genes may affect the chlorophyll content. Therefore, synergistic treatment with different nitrogen forms and PRD may respond to osmotic stress by altering genes that are important in chlorophyll development, metabolism, and synthesis pathways.

## 4 Conclusion

Drought adversely affects rice growth and development, and a PRD and nitrogen cotreatment can alleviate the osmotic stress damage caused by drought by enhancing the SOD activity in leaves to accelerate ROS scavenging, reducing the degree of membrane lipid peroxidation, stabilizing photosynthesis, and enhancing the RWC of leaves. The degree of relief brought about by the cotreatment of different butane forms with PRD was related to different nitrogen forms. Therefore, we performed a comparative analysis of the two vegetative forms and PRD cotreatment and revealed DEGs involved in photosynthesis, nitrogen metabolic processes, phytohormone signaling, and biosynthesis of other secondary metabolites. Some redox-related genes, such as OsGRX4, OsGRX29, OsGRX23, and OsNDPK2, and the metabolites ascorbic acid, GSH, cysteine, and proline may be important in responding to the redox process and mitigating oxidative damage. Some phytohormones and phytohormone-related metabolites undergo complex changes under synergistic treatment with different nitrogen and PRDs, in which the regulation of cytokinin synthesis and signaling pathways are essential. Different nitrogen forms and PRD treatments also altered the expression of two glutamine synthetases, OsGS1;2 and OsGS1;1, and OsNR1.2, which regulate nitrogen metabolism and assimilation. Additionally, some genes involved in photosynthesis responded to osmotic stress by regulating photosynthetic electron transport, chlorophyll development, metabolism, and synthesis pathways.

The results of this study indicate that different nitrogen forms and PRD treatments promote rice leaf development by regulating the expression of different pathway genes to alleviate osmotic stress. This alleviation may be affected by different nitrogen forms. The functions of the candidate DEGs need to be further verified.

## Data availability statement

The original contributions presented in the study are included in the article/**Supplementary Material**, further inquiries can be directed to the corresponding author/s.

## Author contributions

MZ: Conceptualization, Data curation, Visualization, Writing – original draft. ZG: Methodology, Writing – review & editing. CK: Data curation, Writing – original draft. XC: Conceptualization, Funding acquisition, Writing – review & editing.
